# Supplementary material for: Genetic divergences and hybridization within the Sebastes inermis complex
Source: PeerJ. 2023 Nov 15;11:e16391. doi: 10.7717/peerj.16391 (PMC10656903; doi:10.7717/peerj.16391)
Supplement: Supplemental Information 1 [file peerj-11-16391-s001.docx]

| Locus | Repeat Motif | Primer sequence (5’➝3’) | Universal primers | Multiplex | Reference |
| --- | --- | --- | --- | --- | --- |
| SSC12 | AC | F: AACACGCTGAACAGAGAACAAA  R: GCTCCGACTATAGCTGGTCCTA | A | I | Yoshida et al., 2005 |
| Sebi1 | GT | F: TTGTGATTGGTTGGCCTACC  R: GGCGCACAAAAGACTTCTCA | B | I | Blanco Gonzalez et al. 2009 |
| KSs2A | TG | F: TCAACGACCTCGACGACT  R: TCAACGACCTCGACGACT | D | I | An et al. 2009 |
| Sebi3 | GA | F: CTCAGAGTCATGAGCTGGTA  R: CCTGCTGATTTCACCACTAC | A | II | Blanco Gonzalez et al. 2009 |
| SSC23 | TG | F: AGTGTCATGCCCTCTTCCAG  R: CACTCGGCATTCTCACCTCA | A | II | Yoshida et al., 2005 |
| KSs7 | GT | F: TGGGCAATAAATAAGAGAGGA  R: TGGGCAATAAATAAGAGAGGA | B | II | An et al. 2009 |
| Sebi2 | (GT)n(GA)n | F: GACGCCAATACAGCAGCAGA  R: CTGTGCAGCTACCAGATGAT | C | II | Blanco Gonzalez et al. 2009 |
| SRA7-7 | CA | F: GCATGAAAGTGTATGAAAGGC  R: CATGTGATTCTGTGTCTAACTGAG | C | II | Westerman et al. 2005 |
| KSs6 | CA | F: TGCCCAGATACATTTACACAC  R: TAACCCCCACCCACAC | D | II | An et al. 2009 |
| CGN1 | TG | F: GGCCGACCTGATTCACAG  R: TCAACGACCTCGACGACT | D | II | Gao et al. 2017 |
