# Supplementary material for: Genetic divergences and hybridization within the Sebastes inermis complex
Source: PeerJ. 2023 Nov 15;11:e16391. doi: 10.7717/peerj.16391 (PMC10656903; doi:10.7717/peerj.16391)
Supplement: Supplemental Information 2 — N: sample size, HO: observed heterozygosity, HE: expected heterozygosity, and HWE: P value of the statistical to assess deviation from Hardy-Weinberg Equilibrium. Bold values indicate significance in the test. [file peerj-11-16391-s002.docx]

| Sampling location | Locus | SSC12 | Sebi1 | KSs2A | Sebi3 | SSC23 | KSs7 | Sebi2 | SRA7-7 | KSs6 | CGN1 |
| --- | --- | --- | --- | --- | --- | --- | --- | --- | --- | --- | --- |
| Akita,  *S. cheni* (N = 30) | H_O_ | 0.600 | 0.800 | 0.800 | 0.933 | 0.633 | 0.367 | 0.300 | 0.867 | 0.767 | 0.667 |
|  | H_E_ | 0.477 | 0.678 | 0.763 | 0.902 | 0.612 | 0.476 | 0.315 | 0.859 | 0.838 | 0.751 |
|  | HWE | 0.492 | 0.543 | 0.854 | 0.871 | 0.927 | 0.330 | 0.671 | 0.869 | 0.461 | 0.173 |
| Hiroshima,  *S. cheni* (N = 43) | H_O_ | 0.512 | 0.698 | 0.698 | 0.907 | 0.674 | 0.605 | 0.372 | 0.907 | 0.907 | 0.488 |
|  | H_E_ | 0.560 | 0.660 | 0.722 | 0.888 | 0.645 | 0.630 | 0.421 | 0.889 | 0.920 | 0.709 |
|  | HWE | 0.126 | 0.029 | 0.028 | 0.534 | 0.893 | 0.574 | 0.010 | 0.829 | 0.042 | **0.001** |
| Wakayama,  *S. inermis* (N = 37) | H_O_ | 0.649 | 0.730 | 0.649 | 0.946 | 0.784 | 0.541 | 0.703 | 0.784 | 0.784 | 0.757 |
|  | H_E_ | 0.709 | 0.670 | 0.907 | 0.874 | 0.791 | 0.686 | 0.628 | 0.807 | 0.895 | 0.703 |
|  | HWE | 0.077 | 0.492 | **0.001** | 0.255 | 0.396 | 0.109 | 0.516 | 0.274 | 0.173 | 0.203 |
| Hiroshima,  *S. inermis* (N = 42) | H_O_ | 0.714 | 0.524 | 0.762 | 0.762 | 0.905 | 0.667 | 0.548 | 0.786 | 0.952 | 0.619 |
|  | H_E_ | 0.734 | 0.626 | 0.931 | 0.849 | 0.815 | 0.701 | 0.500 | 0.857 | 0.886 | 0.683 |
|  | HWE | 0.965 | 0.051 | **0.000** | 0.075 | 0.888 | 0.758 | 0.522 | 0.664 | 0.422 | 0.341 |
| Kagoshima,  *S. inermis* (N = 32) | H_O_ | 0.750 | 0.656 | 0.781 | 0.813 | 0.813 | 0.656 | 0.406 | 0.813 | 0.938 | 0.563 |
|  | H_E_ | 0.786 | 0.670 | 0.922 | 0.831 | 0.770 | 0.744 | 0.517 | 0.786 | 0.896 | 0.646 |
|  | HWE | 0.117 | 0.233 | 0.010 | 0.982 | 0.211 | 0.295 | 0.087 | 0.828 | 0.154 | **0.001** |
| Wakayama,  *S. ventricosus* (N = 33) | H_O_ | 0.788 | 0.970 | 0.818 | 0.909 | 0.636 | 0.636 | 0.455 | 0.697 | 0.758 | 0.545 |
|  | H_E_ | 0.731 | 0.950 | 0.880 | 0.888 | 0.640 | 0.441 | 0.578 | 0.796 | 0.845 | 0.596 |
|  | HWE | 0.686 | 0.998 | 0.215 | 0.771 | 0.752 | 0.013 | 0.052 | **0.000** | 0.098 | 0.252 |
| Hiroshima,  *S. ventricosus* (N = 41) | H_O_ | 0.732 | 0.927 | 0.683 | 0.902 | 0.659 | 0.537 | 0.512 | 0.854 | 0.780 | 0.439 |
|  | H_E_ | 0.691 | 0.968 | 0.858 | 0.876 | 0.736 | 0.456 | 0.552 | 0.860 | 0.788 | 0.626 |
|  | HWE | 0.735 | 0.542 | 0.011 | 0.818 | 0.120 | 0.063 | 0.231 | 0.544 | 0.675 | **0.001** |
| Kagoshima,  *S. ventricosus* (N = 28) | H_O_ | 0.643 | 0.964 | 0.857 | 0.929 | 0.821 | 0.857 | 0.536 | 0.857 | 0.750 | 0.464 |
|  | H_E_ | 0.716 | 0.965 | 0.927 | 0.908 | 0.775 | 0.568 | 0.514 | 0.784 | 0.880 | 0.529 |
|  | HWE | 0.779 | 0.778 | 0.205 | 0.094 | 0.925 | **0.001** | 0.407 | 0.987 | 0.094 | 0.256 |
