# Supplementary material for: Genetic divergences and hybridization within the Sebastes inermis complex
Source: PeerJ. 2023 Nov 15;11:e16391. doi: 10.7717/peerj.16391 (PMC10656903; doi:10.7717/peerj.16391)
Supplement: Supplemental Information 3 — The adjusted P value was calculated using the False Discovery Rate method (Benjamini & Hochberg, 1995). α indicates the level of divergent selection of the locus, while FST is the genetic distance between species. Bold values indicate significance of the corrected P values. [file peerj-11-16391-s003.docx]

| Loci | Probability | Adjusted *P* value | α | FST |
| --- | --- | --- | --- | --- |
| SSC12 | 0.044 | 0.559 | 0.002 | 0.065 |
| Sebi1 | 0.275 | 0.246 | -0.116 | 0.058 |
| KSs2A | 0.228 | 0.351 | -0.102 | 0.059 |
| Sebi3 | 0.161 | 0.432 | -0.089 | 0.059 |
| SSC23 | 0.074 | 0.503 | -0.038 | 0.063 |
| KSs7 | 0.993 | **0.004** | **1.380** | 0.215 |
| Sebi2 | 0.750 | 0.086 | 0.898 | 0.156 |
| SRA7-7 | 0.037 | 0.604 | 0.003 | 0.065 |
| KSs6 | 0.035 | 0.640 | -0.009 | 0.065 |
| CGN1 | 0.998 | **0.002** | **1.496** | 0.233 |
