# Supplementary material for: Genetic divergences and hybridization within the Sebastes inermis complex
Source: PeerJ. 2023 Nov 15;11:e16391. doi: 10.7717/peerj.16391 (PMC10656903; doi:10.7717/peerj.16391)
Supplement: Supplemental Information 4 — N: sample size, Nh: number of haplotypes, H: haplotype diversity, and π: nucleotide diversity. [file peerj-11-16391-s004.docx]

|  | N | Nh | Hd | Nd |
| --- | --- | --- | --- | --- |
| *S. cheni* | 25 | 19 | 0.97 | 0.071 |
| *S. inermis* | 25 | 18 | 0.957 | 0.106 |
| *S. ventricosus* | 25 | 14 | 0.903 | 0.055 |
| Black-white | 22 | 18 | 0.978 | 0.094 |
| Red-white | 19 | 18 | 0.994 | 0.077 |
