# Supplementary material for: Genetic divergences and hybridization within the Sebastes inermis complex
Source: PeerJ. 2023 Nov 15;11:e16391. doi: 10.7717/peerj.16391 (PMC10656903; doi:10.7717/peerj.16391)
Supplement: Supplemental Information 5 — Positions were shaded in grey-scale colours indicating whether they occur only in the Sebastes inermis complex (light grey) or are only present in one species within the complex (dark grey). Most of these positions have been proved to be under selection in the genus (119, 158, 205, 213, 217, 274, and 277) (Sivasundar & Palumbi, 2010), near the chromophore (133) (Sugawara et al., 2005) or are functionally important in spectral tunning (165). This table was adapted from Sivasundar & Palumbi (2010). [file peerj-11-16391-s005.docx]

|  | **Amino acid position** | | | | | | | | | |  |
| --- | --- | --- | --- | --- | --- | --- | --- | --- | --- | --- | --- |
|  | **1** | **1** | **1** | **1** | **2** | **2** | **2** | **2** | **2** | **2** | |
|  | **1** | **3** | **5** | **6** | **0** | **1** | **1** | **7** | **7** | **8** | |
| Species | **9** | **3** | **8** | **5** | **5** | **3** | **7** | **4** | **7** | **6** | |
| ***S. cheni*** | **L** | **I** | **A** | **A** | **I** | **A** | **M** | **F** | **L** | **I** | |
| ***S. inermis*** | **.** | **.** | **.** | **S** | **.** | **.** | **.** | **.** | **.** | **.** | |
| ***S. ventricosus*** | **.** | **.** | **.** | **S** | **.** | **.** | **.** | **.** | **.** | **.** | |
| **"Kumano"** | **.** | **.** | **.** | **S** | **.** | **.** | **T** | **.** | **.** | **.** | |
| ***S. alutus*** | **.** | **V** | **G** | **C** | **.** | **S** | **T** | **.** | **.** | **V** | |
| ***S. auriculatus*** | **.** | **V** | **.** | **S** | **.** | **.** | **.** | **Y** | **S** | **V** | |
| ***S. aurora*** | **I** | **V** | **G** | **C** | **V** | **S** | **T** | **Y** | **S** | **V** | |
| ***S. brevispinis*** | **.** | **V** | **G** | **C** | **V** | **S** | **T** | **Y** | **S** | **V** | |
| ***S. carnatus*** | **.** | **V** | **.** | **S** | **.** | **.** | **.** | **Y** | **S** | **V** | |
| ***S. caurinus*** | **.** | **V** | **.** | **S** | **.** | **.** | **.** | **Y** | **S** | **V** | |
| ***S. chlorostictus*** | **I** | **V** | **G** | **C** | **V** | **S** | **T** | **Y** | **C** | **V** | |
| ***S. chrysomelas*** | **.** | **V** | **.** | **S** | **.** | **.** | **.** | **Y** | **S** | **V** | |
| ***S. ciliatus*** | **.** | **V** | **G** | **C** | **.** | **S** | **T** | **.** | **.** | **V** | |
| ***S. constellatus*** | **.** | **V** | **G** | **C** | **V** | **S** | **T** | **Y** | **F** | **V** | |
| ***S. diploproa*** | **V** | **V** | **G** | **C** | **V** | **S** | **T** | **Y** | **S** | **V** | |
| ***S. elongatus*** | **I** | **V** | **G** | **C** | **V** | **S** | **T** | **Y** | **S** | **V** | |
| ***S. entomelas*** | **.** | **V** | **G** | **C** | **V** | **S** | **T** | **Y** | **S** | **V** | |
| ***S. flavidus*** | **.** | **V** | **G** | **C** | **V** | **S** | **.** | **.** | **S** | **V** | |
| ***S. hopkinsi*** | **.** | **V** | **G** | **C** | **V** | **S** | **T** | **Y** | **S** | **V** | |
| ***S. maliger*** | **.** | **V** | **G** | **C** | **.** | **S** | **.** | **Y** | **S** | **V** | |
| ***S. marinus*** | **I** | **V** | **G** | **C** | **.** | **S** | **T** | **.** | **.** | **V** | |
| ***S. melanops*** | **.** | **V** | **G** | **C** | **V** | **F** | **V** | **.** | **S** | **V** | |
| ***S. melanostomus*** | **I** | **V** | **G** | **C** | **V** | **S** | **T** | **Y** | **S** | **V** | |
| ***S. mentella* "deep"** | **I** | **V** | **G** | **C** | **.** | **S** | **T** | **.** | **.** | **V** | |
| ***S. mentella* "shallow"** | **V** | **V** | **G** | **C** | **.** | **S** | **T** | **.** | **.** | **V** | |
| ***S. miniatus*** | **.** | **V** | **.** | **C** | **.** | **S** | **.** | **Y** | **S** | **V** | |
| ***S. mystinus*** | **.** | **V** | **G** | **C** | **V** | **S** | **.** | **Y** | **S** | **V** | |
| ***S. nebulosus*** | **.** | **V** | **.** | **S** | **.** | **T** | **.** | **Y** | **S** | **V** | |
| ***S. nigrocinctus*** | **.** | **V** | **G** | **C** | **V** | **S** | **T** | **Y** | **S** | **V** | |
| ***S. ovalis*** | **.** | **V** | **G** | **C** | **V** | **S** | **T** | **Y** | **S** | **V** | |
| ***S. paucispinis*** | **.** | **V** | **G** | **C** | **V** | **F** | **.** | **Y** | **S** | **V** | |
| ***S. pinniger*** | **.** | **V** | **G** | **C** | **.** | **S** | **.** | **Y** | **S** | **V** | |
| ***S. proriger*** | **.** | **V** | **G** | **C** | **V** | **S** | **T** | **Y** | **S** | **V** | |
| ***S. rosaceus*** | **.** | **V** | **G** | **C** | **V** | **S** | **T** | **Y** | **F** | **V** | |
| ***S. ruberrimus*** | **.** | **V** | **G** | **C** | **.** | **S** | **.** | **Y** | **S** | **V** | |
| ***S. rubrivinctus*** | **.** | **V** | **G** | **C** | **V** | **S** | **T** | **Y** | **S** | **V** | |
| ***S. semicinctus*** | **.** | **V** | **G** | **C** | **V** | **S** | **T** | **Y** | **S** | **V** | |
| ***S. serranoides*** | **.** | **V** | **G** | **C** | **V** | **F** | **V** | **.** | **S** | **V** | |
| ***S. umbrosus*** | **.** | **V** | **G** | **C** | **V** | **S** | **T** | **Y** | **F** | **V** | |
| ***S. viviparus*** | **I** | **V** | **G** | **C** | **.** | **S** | **T** | **.** | **.** | **V** | |
