# Supplementary figures and images for: Genetic divergences and hybridization within the Sebastes inermis complex
Source: PeerJ. 2023 Nov 15;11:e16391. doi: 10.7717/peerj.16391 (PMC10656903; doi:10.7717/peerj.16391)

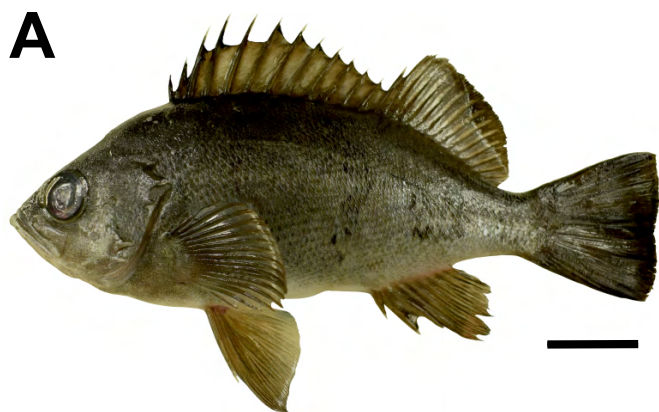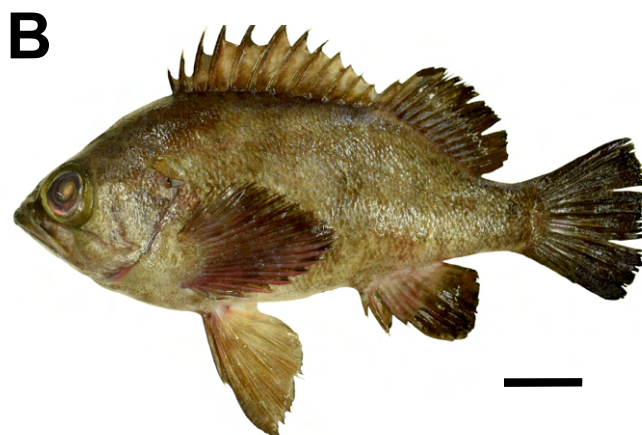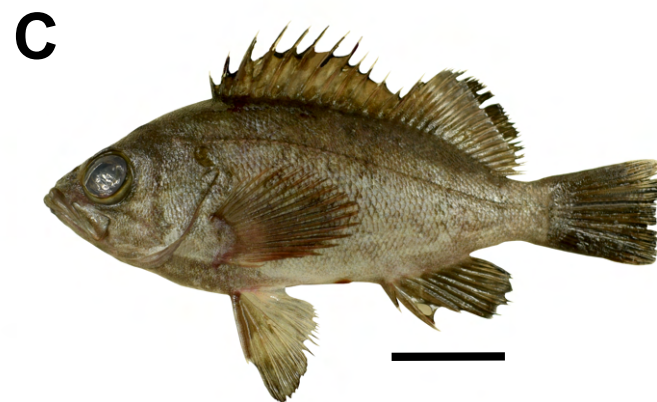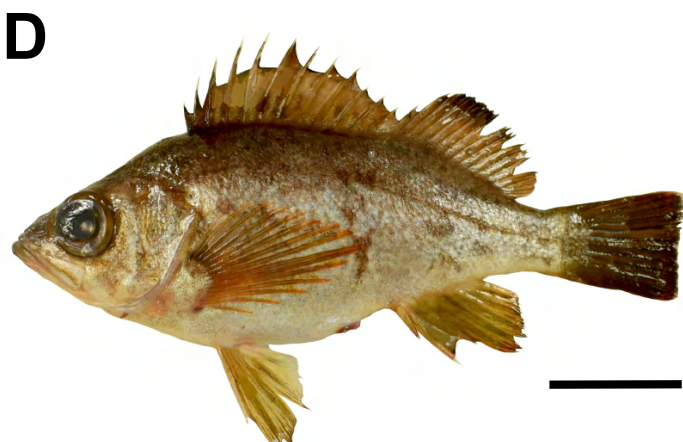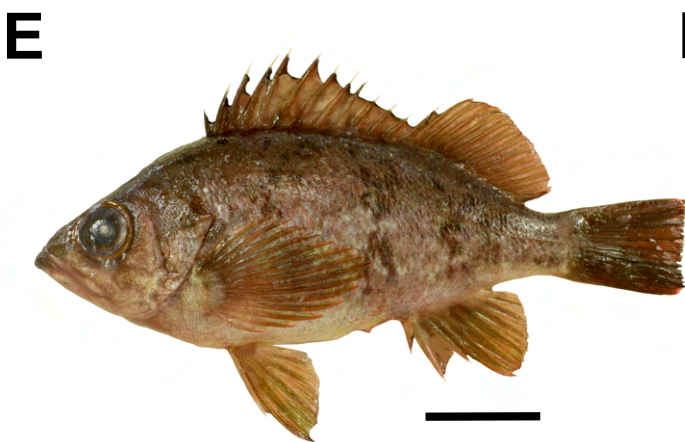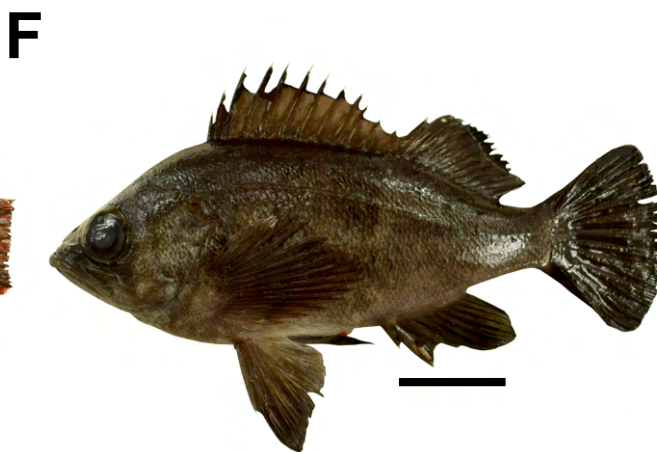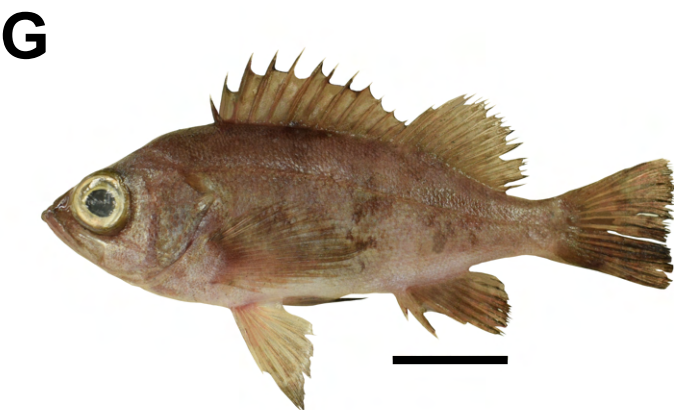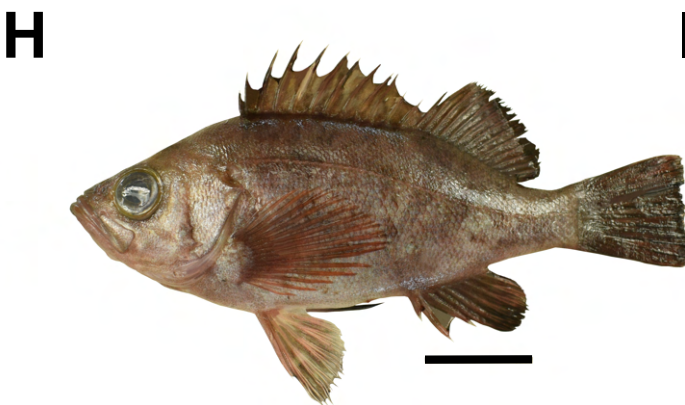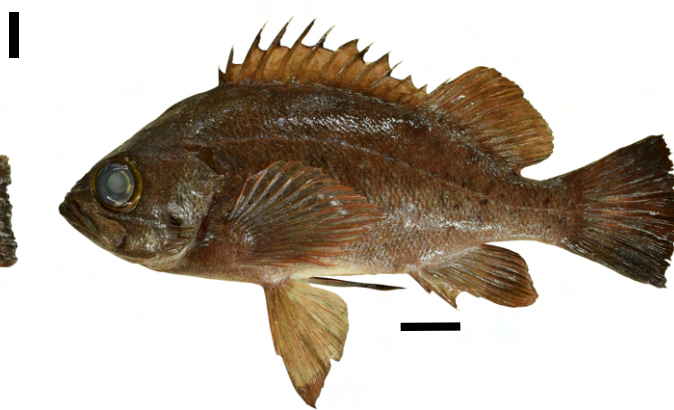

Supplement: Supplemental Information 6 — (A) black rockfish (Sebastes ventricosus), (B and C) white rockfish (Sebastes cheni), (D and E) red rockfish (Sebastes inermis), (F) black-withe PMH, (G and H), red-white PMH, and (I) “Kumano” PMH. Black horizontal lines represent size scales of 3 cm. [file peerj-11-16391-s006.pdf]

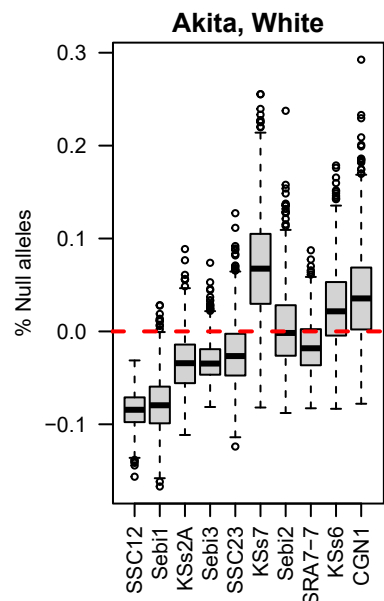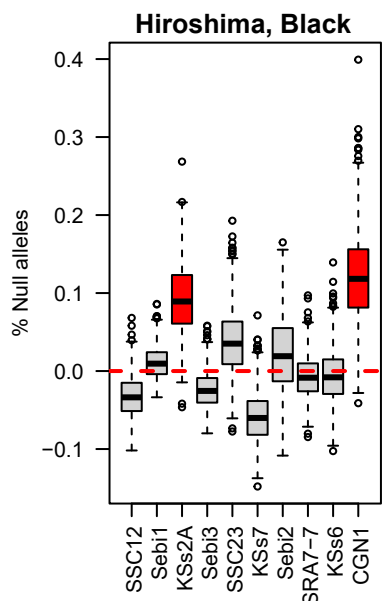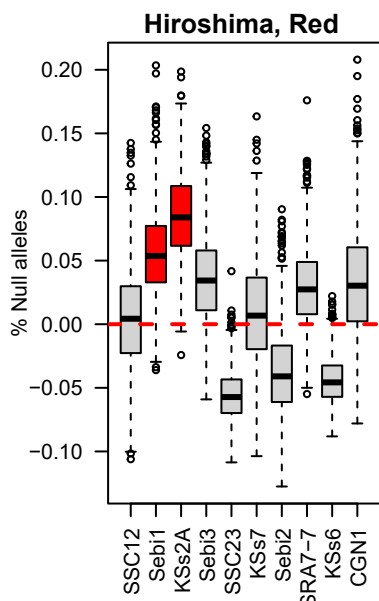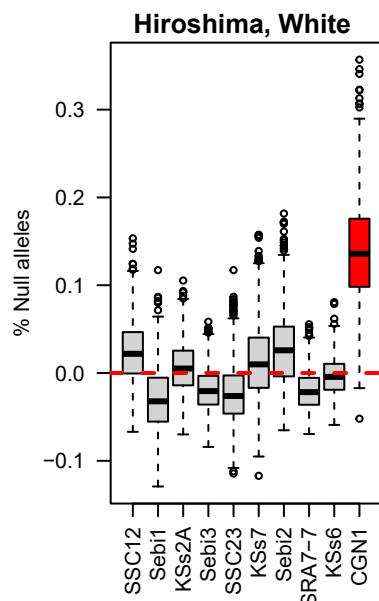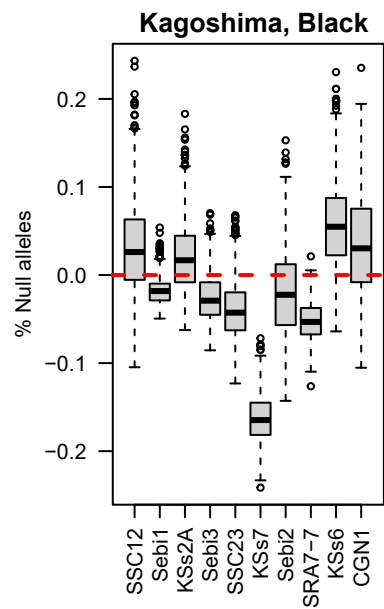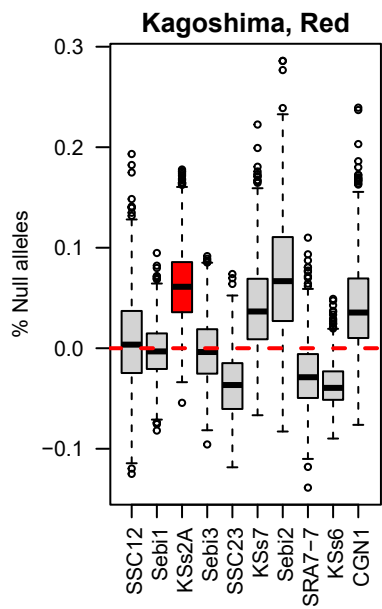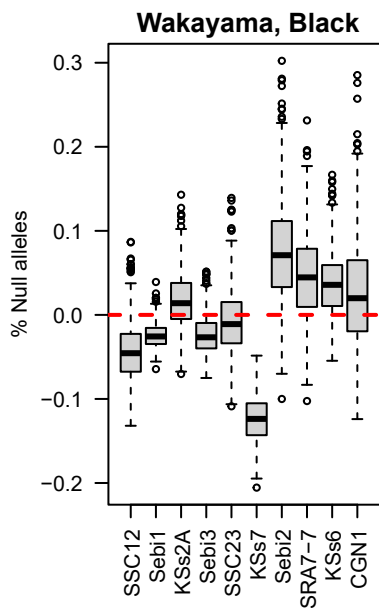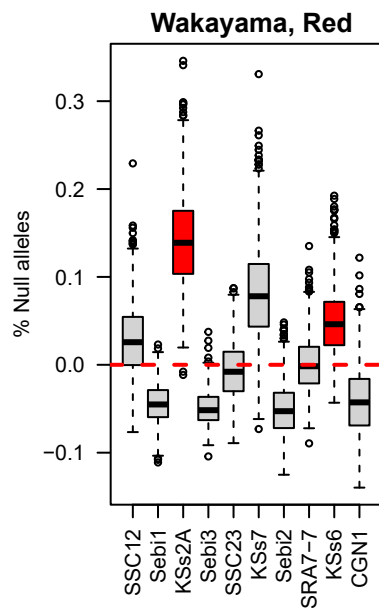

Supplement: Supplemental Information 7 — Title of each plot indicates the sampling location and species (black, red, or white rockfish). Red boxes indicate a significant presence of null alleles detected by Micro-Checker. Red lines indicated 0% of null alleles. [file peerj-11-16391-s007.pdf]

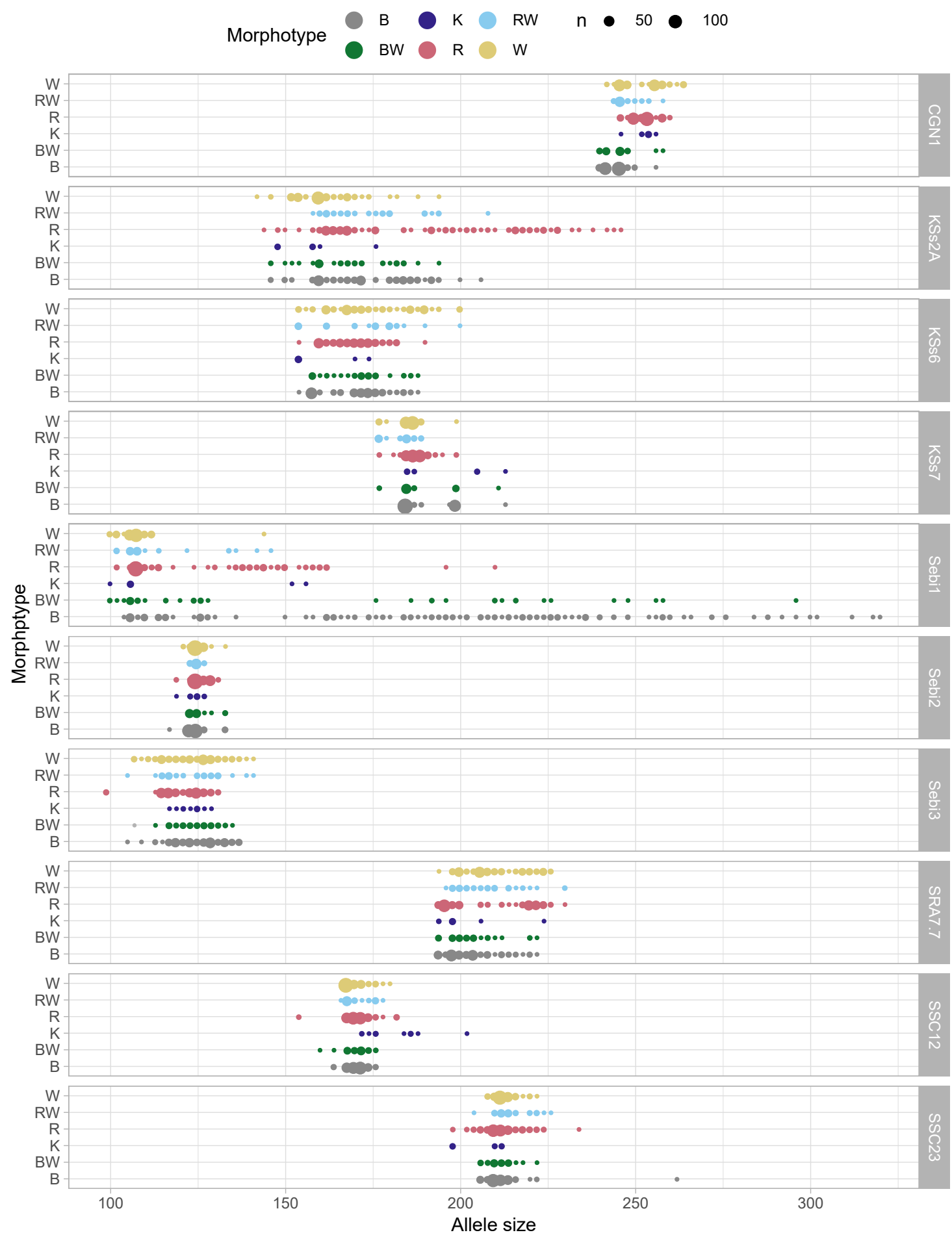

Supplement: Supplemental Information 8 — B: black rockfish S. ventricosus, R: red rockfish S. inermis, W: white rockfish S. cheni, BW: black-white morphotype, RW: red-white morphotype, and K: “Kumano” morphotype. [file peerj-11-16391-s008.pdf]

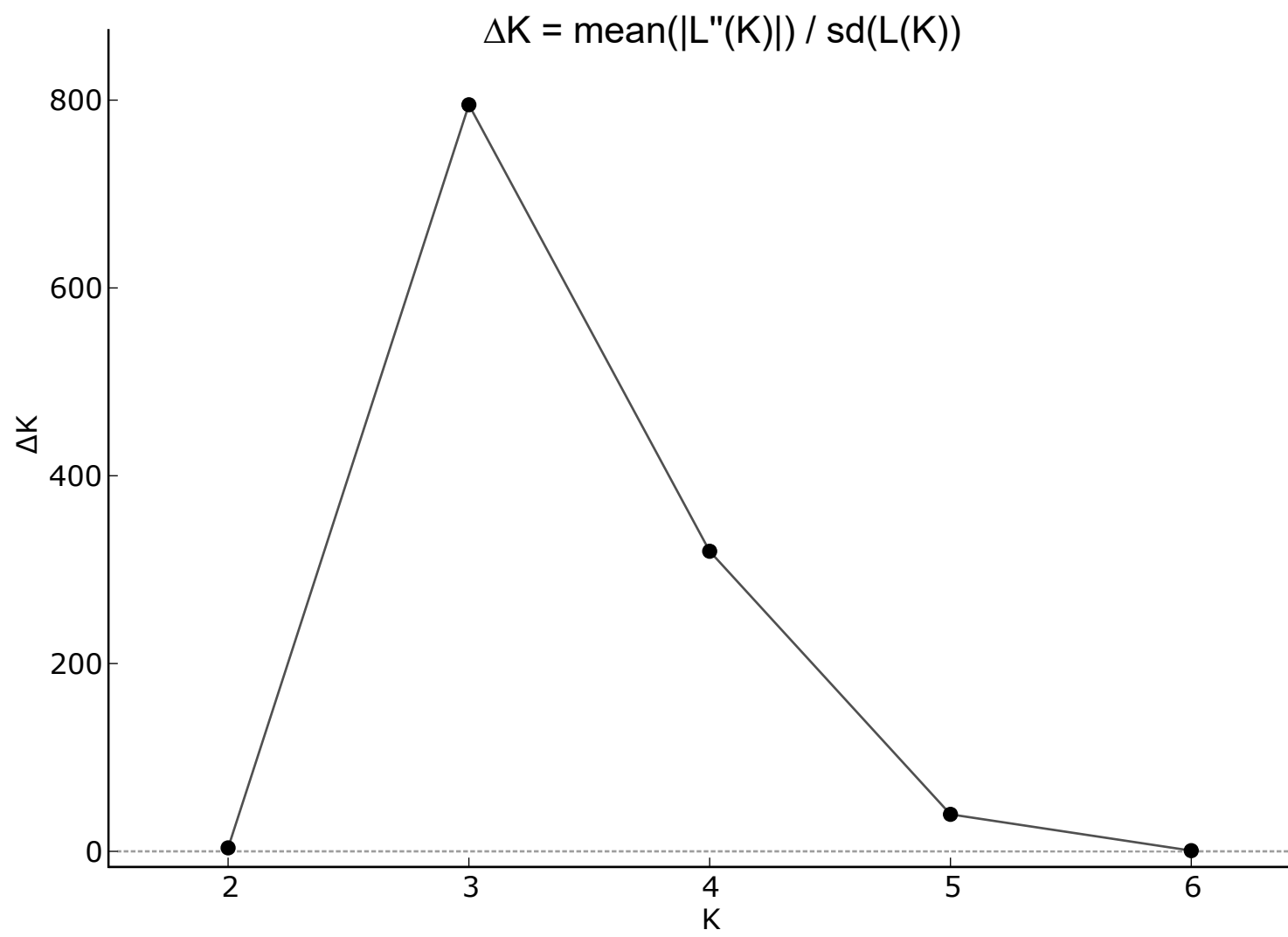

Supplement: Supplemental Information 9 [file peerj-11-16391-s009.pdf]

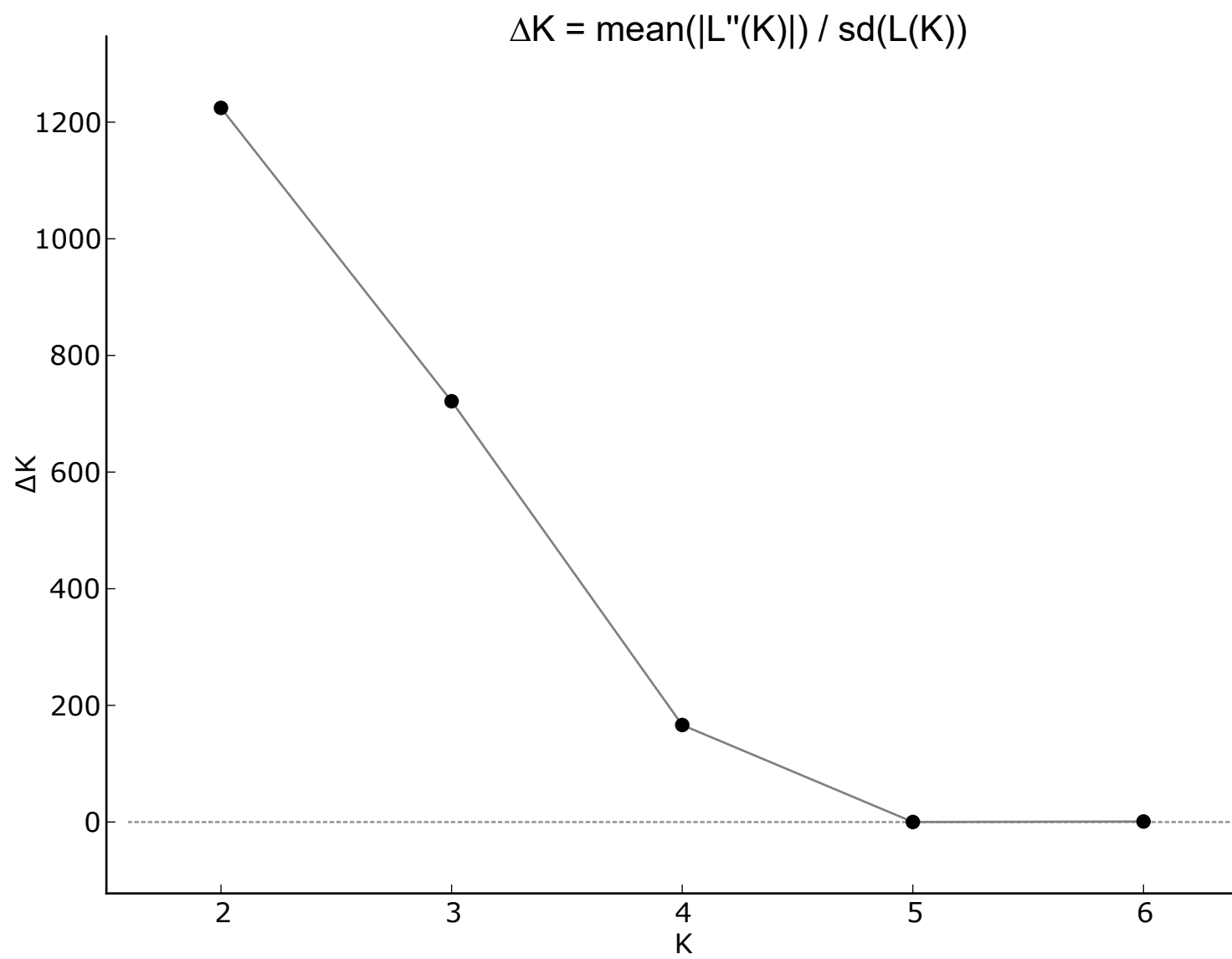

Supplement: Supplemental Information 10 [file peerj-11-16391-s010.pdf]
